# Effect of sevoflurane on hemodynamic response during cardiopulmonary bypass in cardiac surgery patients: A randomized controlled trial

**DOI:** 10.34172/jcvtr.025.33239

**Published:** 2025-09-28

**Authors:** Vu Thanh Lam, Nguyen Minh Ly

**Affiliations:** ^1^Department of Anesthesiology and Pain Management, Vinmec Times City International Hospital, Hanoi, Vietnam; ^2^Department of Anesthesia and Critical Care, 108 Military Central Hospital, Hanoi, Vietnam

**Keywords:** Bypass, Cardiopulmonary, Hemodynamics, Anesthesia, Thoracic surgery

## Abstract

**Introduction:**

Sevoflurane has little effect on hemodynamics and has been shown to have a hemodynamic stabilizing effect in the pre- and post-cardiopulmonary bypass (CPB) period in patients undergoing cardiac surgery. However, clinical data on the effect of sevoflurane on the hemodynamic response during CPB in patients undergoing cardiac surgery are lacking. This study investigated whether the hemodynamic stabilizing effect of sevoflurane is demonstrated during CPB time in cardiac surgery patients.

**Methods:**

Fifty-five patients undergoing cardiac surgery under CPB were randomly assigned to anesthesia with sevoflurane (intervention group) or propofol (control group) during CPB. The primary outcomes were changes in hemodynamic parameters and the need for inotropes and vasopressors during CPB. Secondary outcomes were morbidity and mortality within 30 days after surgery.

**Results:**

The mean arterial pressure (MAP) at 5 minutes after heartbeat recovery and the end of CPB as well as central venous oxygen saturation (ScvO_2_) at 5 minutes after heartbeat recovery and cardiac index (CI) at the end of CPB of group S-CPB (intervention group) were higher than those of group P-CPB (control group). In addition, the proportion of patients using dobutamine and noradrenaline during CPB was also lower in group S-CPB.

**Conclusion:**

In conclusion, in patients undergoing cardiac surgery under CPB, the use of sevoflurane for anesthesia during CPB results in hemodynamic stability with less need for inotropes and vasopressors during CPB but morbidity and mortality within 30 days after surgery were not significantly different when compared with the control group.

## Introduction

 Cardiopulmonary bypass (CPB) is frequently used for cardiac surgery to temporarily replace or support heart and lung function.^1-3^ Despite significant advances in techniques, CPB still causes many unpredictable complications for patients.^1,4^ Maintaining stable hemodynamics during CPB will be a challenge not only for the cardiac surgeon and the perfusionist but also for the cardiac anesthesiologist, which will contribute to reducing morbidity and mortality. Sevoflurane and propofol are the most commonly used anesthetics for anesthesia before, during and after CPB in patients undergoing cardiac surgery under CPB due to their effectiveness and safety in anesthesia and especially their myocardial protective and hemodynamically stable properties.^5-11^ However, in the past in Vietnam, during the CPB stage, because the cardiopulmonary bypass circuit was not equipped with a vaporizer, intravenous anesthetics such as propofol were often used to maintain anesthesia for the patient. Recently, cardiopulmonary bypass circuits have been equipped with sevoflurane vaporizers. An issue we are interested in is whether hemodynamics during CPB with sevoflurane anesthesia are more stable when compared with intravenous anesthetics in cardiac surgery patients in Vietnam. Some studies around the world have only mentioned the myocardial protective properties and effects on general hemodynamics of sevoflurane when compared with intravenous anesthetics in patients undergoing coronary artery bypass surgery and heart valve surgery under CPB and showed inconsistent results regarding the advantage of sevoflurane versus intravenous anesthetics.^7-14^ Currently, during CPB time, only a few studies have addressed the hemodynamic effects of sevoflurane when compared with propofol in patients undergoing CABG surgery under CPB, but the results are still controversial and no studies have addressed this issue in patients undergoing valvular and congenital heart surgery under CPB.^15-17^ Therefore, we designed a randomized controlled clinical intervention study to evaluate the effect of sevoflurane on hemodynamic response during CPB in cardiac surgery patients in Vietnam including patients undergoing heart valve surgery and congenital heart surgery (atrial septal defect repair, ventricular septal defect repair). The primary objective of this study was to determine whether, compared with propofol, sevoflurane anesthesia during CPB time has a greater effect on hemodynamic parameters and vasopressor requirements during CPB. Because hemodynamic instability increases the risk of postoperative complications and mortality^18-20^, the secondary objective was to evaluate this effect of sevoflurane on complications and mortality within 30 days after surgery.

## Materials and Methods

###  Patient Population

 This was a randomized controlled clinical intervention study conducted at 108 Military Central Hospital, Vietnam between April 2016 and December 2019. We selected patients aged 18 years and older who were scheduled to undergo elective cardiac surgery under CPB, including heart valve surgery and congenital heart surgery (atrial septal defect repair, ventricular septal defect repair). Patients with coronary artery disease with unstable angina or confirmed by coronary angiography had reported in several studies^15,16,17^ and were therefore excluded from the design of this study. Because the 5-6 week period after myocardial infarction is the time required for healing of the infarcted myocardium, patients who had an acute myocardial infarction within the previous 6 weeks were also excluded.^21^ In addition, we also excluded patients who underwent previous cardiac surgery, preoperative left ventricular ejection fraction (LVEF) < 40%, preoperative inotropic or vasopressor or balloon therapy, severe chronic obstructive pulmonary disease (forced expired volume in 1 s < 0.8 L), hepatic disease (serum alanine aminotransferase or aspartate aminotransferase concentration > 150 IU L^-1^), renal insufficiency (serum creatinine concentration > 1.5 mg dL^-1^), history of drug allergy or malignant hyperthermia, history of nervous system diseases or psychiatric disturbance, pregnancy, withdrawal of consent and reoperation.

 The ethics committee of 108 Military Central Hospital approved this study design (ref. 275/QD-V108) and written informed patient consent was obtained. The study was conducted according to the Declaration of Helsinki.

###  Study Groups

 The patients were randomly assigned to group S-CPB (intervention group) or group P-CPB (control group) with equal size according to computer-generated randomization. The patients in group P-CPB were anesthetized with a target-controlled propofol infusion throughout the entire anesthetic procedure (induction and maintenance of anesthesia before, during and after CPB), while the patients in group S-CPB received sevoflurane as an alternative to propofol during CPB time. A computer-generated random code determined which anesthetic protocol was identified by each treatment number. Subjects were assigned the treatment numbers in ascending chronological order of admission in the study. The participant randomization assignment was concealed in an envelope until the start of anesthesia. The surgeons, research assistants, and medical and nursing staff in the intensive care unit and the ward were blinded to the study.

###  Primary and Secondary End-Points

 The primary endpoints were the change in hemodynamic parameters (mean arterial blood pressure, cardiac index, systemic vascular resistance index, central venous oxygen saturation) and the need for inotropes and vasopressors during CPB. Monitoring of hemodynamic parameters was performed with the FloTrac/EV1000 platform. The FloTrac sensor was connected to a standard radial artery catheter and was used to calculate stroke volume based on analysis of the arterial pressure waveform and patient physiology, and from this value, cardiac output and other values were determined. The FloTrac/EV1000 platform worked well during the beating - heart phase of the cardiopulmonary bypass. It was also connected to a central venous pressure catheter to calculate systemic vascular resistance and a PreSep Oximetry Catheter to measure venous oxygen saturation of the superior vena cava.^22,23^ In addition, blood lactate, a marker of tissue perfusion that is influenced not only by the macrocirculation but also by the microcirculation, was also assessed. Secondary endpoints were morbidity and mortality rates within 30 days after surgery.

###  Anesthetic Protocols 

 All patients received midazolam of 0.04 mg kg^-1^ intravenously 30 minutes before induction of anesthesia as premedication. In both groups, anesthesia was induced with a target-controlled infusion (TCI) of fentanyl at 2 ng mL^-1^ and of propofol at 1.5 μg mL^-1^, increased 0.5 μg mL^-1^ every two minutes if patients have not lost consciousness). Next, muscle paralysis was obtained with 0.1 mg kg^-1^ pipecuronium bromide to facilitate tracheal intubation. Mechanical ventilation was adjusted in assist-control mode with a tidal volume of 6–8 mL kg^-1^ body weight, respiratory frequency was adjusted to obtain an end-tidal carbon dioxide pressure of 35–45 mmHg, inspired oxygen fraction was set at 0.5 and positive end-expiratory pressure of 5 cmH_2_O was set as default. Then, in both groups, anesthesia was maintained with TCI of fentanyl at 2 ng mL^-1^, TCI of propofol at 3 – 4 μg mL^-1^ and pipecuronium bromide at 0.04 mg kg^-1^ every 2 hours until the start of CPB. Afterwards, group P-CPB (control group) continued to maintain the protocol throughout the CPB time. As for group S-CPB (intervention group), propofol was replaced by sevoflurane at 1 ± 0.2 minimum alveolar concentration (MAC) throughout the CPB time. At the end of CPB, in both groups, anesthesia was maintained with fentanyl, propofol and pipecuronium bromide according to the protocol as above. During CPB, sevoflurane was administered through the oxygenator. In both groups, the depth of anesthesia before, during and after CPB was controlled at Bispectral index (BIS) 40 – 60 by adjusted inhaled sevoflurane concentration or the infusion rate of propofol, respectively.

###  Perioperative Procedure

 Standard intraoperative monitoring consists of five-lead electrocardiography, a left radial artery catheter, central venous pressure, pulse oxygen saturation, end-tidal carbon dioxide pressure, esophageal temperature monitoring and urine output. Additionally, Bispectral index (BIS) monitoring (which measures depth of anesthesia), hemodynamic monitoring (with the FloTrac/EV1000 platform), and transesophageal echocardiography were also performed in all patients. Routine surgical and CPB techniques were used in all patients of two groups by a same group of cardiac surgeons. Before initializing CPB, systemic heparinization was accomplished with a heparin dose of 300 IU kg^-1^. Additional heparin was administered during CPB to maintain an activated coagulation time of > 400 s. CPB was maintained at a cardiac index of 2.4 L min^-1^ m^-2^ of body surface area with a Sarns heart–lung roller pump (Terumo CV Systems, Ann Arbor, MI, USA). The pump prime consisted of 1,000 ml lactated Ringer’s solution and 100 ml 20% mannitol, to which 10,000 U heparin was added. The mean arterial blood pressure (MAP) was maintained at more than 65 mmHg by increasing the pump flow rate or a bolus of phenylephrine (100 μg) or norepinephrine (5 μg). Surgery was performed under normal body temperature (36 - 37°C). After aortic cross-clamping, cardioplegia was achieved with the warm blood solution administered into the aortic root every 30 minutes, according to a standard protocol. After aortic unclamping, the heart was defibrillated if sinus rhythm does not resume spontaneously. Serum glucose levels were controlled with intermittent administration of insulin (intravenous bolus of 5 –10 UI). Patients with a hemoglobin value below 8 g dL^-1^ received homologous red blood cell transfusions. At the end of CPB, protamine was administered as required to return the ACT to the baseline values (1 mg protamine for 100 IU heparin). Patients were transferred to the intensive care unit (ICU) where they were sedated with midazolam/fentanyl and extubated when pressure support ventilation is tolerated.

 In this study, hypotension was defined as a mean arterial blood pressure < 65 mmHg. Inotropic and vasopressor drugs were administered by continuous infusion if CI was low ( < 2.4 L min^-1^ m^-2^), depending on whether afterload was high, normal or low (normal SVRI 1700–2400 dyn s cm^-5^ m^-2^).^24^ Dobutamine was started at 2 μg kg^-1^ min^-1^ and was titrated up to 10 μg kg^-1^ min^-1^. In the case of clinically significant tachycardia (HR > 110 min^-1^ or > 15% increase over predosing values), dobutamine was decreased. If dobutamine was ineffective, milrinone was started with a bolus administration (50 μg kg^-1^ over 10 min), and was continued with dose 0.375–0.5 μg kg^-1^ min^-1^. When both dobutamine and milrinone were ineffective, epinephrine was started at 0.04 μg kg^-1^ min^-1^. The epinephrine dose rate was titrated up to 0.1 μg kg^-1^ min^-1^. Norepinephrine was started at 0.04 μg kg^-1^ min^-1^ and was titrated up to 0.5 μg kg^-1^ min^-1^. If hypotension was not responsive to norepinephrine, vasopressin was combined with norepinephrine, with a starting dose rate of 1 IU h^-1^. The dose rate was titrated up to 4 IU h^-1^.^25^

###  Data Collection

 Hemodynamic data including mean arterial blood pressure (MAP), cardiac index (CI), systemic vascular resistance index (SVRI), central venous oxygen saturation (ScvO2) and plasma lactate levels were recorded just before the start of CPB (T_1_), 5 minutes after heartbeat recovery (T_2_) and the end of CPB (T_3_).

###  Statistical Analysis

 Regarding the sample size of the study, we considered CI at the end of CPB as the primary endpoint. Based on a pilot study with 7 cases in each group, the expected mean CI was 2.8 ± 0.5 L min^-1^ m^-2^ in group S-CPB and 2.4 ± 0.6 L min^-1^ m^-2^ in group P-CPB. For a power of 0.8 and α = 0.05, based on the formula estimating sample size for comparison of two means,^26^ we calculated that a sample size of at least 26 patients in each group was appropriate.

 Qualitative variables were described by frequency and percentage, were compared using the χ2 test or Fisher exact test as appropriate. Quantitative variables were presented as mean ± standard deviation (SD) or median (25–75%, interquartile range) and were compared by the Student’s t-test or the Mann-Whitney U test as appropriate. All data were analyzed by medical statistics algorithm with SPSS 26.0 software (SPSS Inc, Chicago, IL, USA) and statistical significance was accepted at *P* < 0.05. All reported *P* values were two-tailed.

## Results

**Figure 1:**
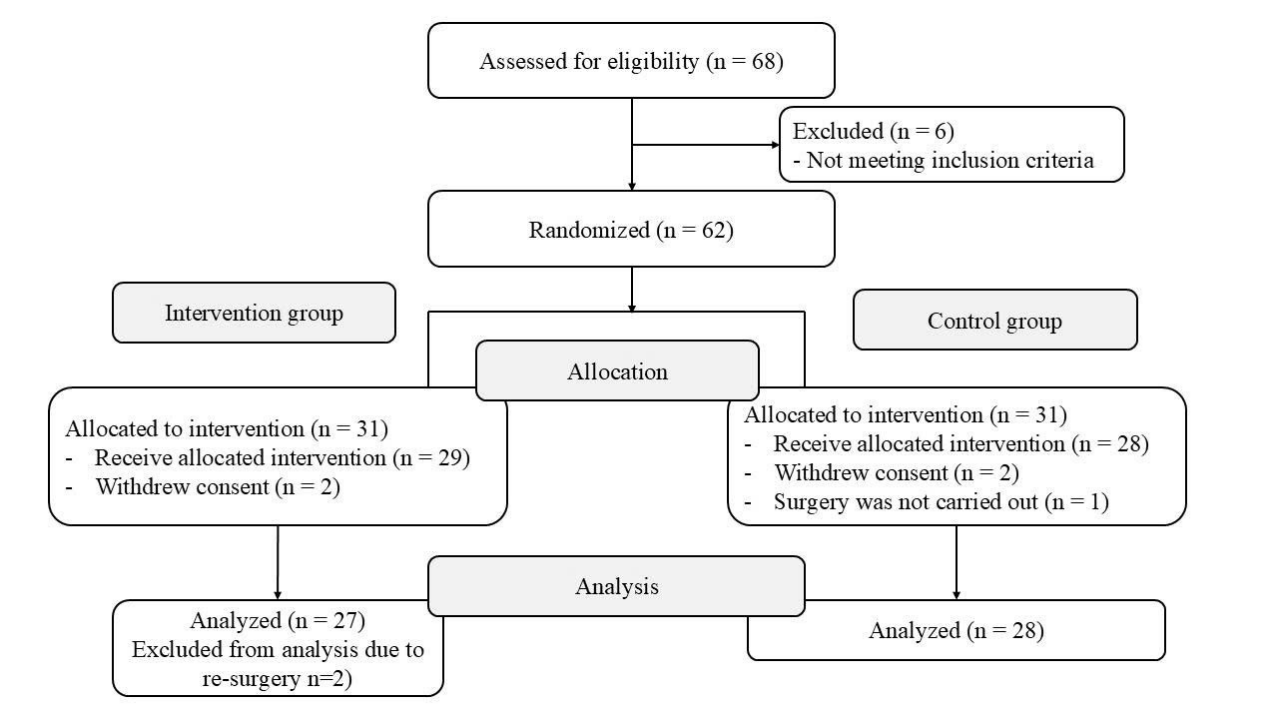
CONSORT flow diagram

 From April 2016 through December 2019, a total of 62 patients were randomized. One was excluded because surgery was not carried out, four withdrew consent and two had to have re-surgery. Of the remaining 55 patients, 27 had been allocated to group S-CPB (intervention group) and 28 to group P-CPB (control group). The flow diagram is shown in Figure 1. The characteristics of the two groups were similar (Table 1).

**Table 1:** Patient’s characteristics

**Patient characteristics**	**Group S-CPB (** ***n*** **=27)**	**Group P-CPB (** ***n*** **=28)**	***P*** ** value**
Preoperative data			
Age (year)	50.5 ± 11.4	49.1 ± 15.1	0.698
Sex (M/F)	14/13	16/12	0.694
Weight (kg)	51.8 ± 6.6	54.3 ± 7.6	0.205
Height (cm)	160.0 ± 5.5	162.3 ± 7.1	0.199
BMI (g.m^-2^)	20.2 ± 2.1	20.6 ± 2.2	0.520
ASA class (II/III/IV)	7/19/1	11/17/0	0.391
NYHA (I/II/III)	2/18/7	1/22/5	0.556
EF (%)	64.2 ± 12.4	63.2 ± 8.6	0.747
SPAP (mmHg)	45.7 ± 25.7	44.7 ± 17.4	0.862
Euro SCORE II	1.3 ± 0.6	1.4 ± 1.1	0.761
Diabetes (n, %)	0 (0.0)	0 (0.0)	NA
Hypertension (n, %)	3 (11.1)	3 (10.7)	1.000
Types of surgery (n, %)			
Replace/repair the mitral valve	11 (40.7)	15 (53.6)	0.341
Replace the aortic valve	2 (7.4)	2 (7.1)	1.000
Replace/repair the mitral valve and replace the aortic valve	5 (18.5)	3 (10.7)	0.469
Replace/repair the mitral valve and shaping of tricuspid valve	7 (25.9)	5 (17.9)	0.469
Patching the atrial/ventricular septal defect	2 (7.4)	3 (10.7)	1.000
Intraoperative data			
Midazolam (mg)	2.0 ± 0.1	2.0 ± 0.2	0.986
Fentanyl (mg)	0.9 ± 0.2	1.0 ± 0.3	0.511
Pipecuronium (mg)	8.2 ± 1.3	8.5 ± 1.4	0.382
Anesthesia time (min)	239.4 ± 33.2	238.2 ± 41.0	0.903
Operating time (min)	198.7 ± 34.2	199.1 ± 41.1	0.971
Aortic clamp time (min)	70.7 ± 24.7	71.7 ± 30.3	0.893
CPB time (min)	91.8 ± 30.2	95.4 ± 32.8	0.675
CPB withdrawal time (min)	16.5 ± 6.8	18.1 ± 6.5	0.377
Flow rate (L min^-1^)	4.6 ± 0.3	4.8 ± 0.4	0.100

Data are presented as mean ± SD, unless noted otherwise. BMI = Body mass index; ASA class = American Society of Anesthesiologists physical status classification; NYHA = New York Heart Association; EF = Ejection fraction; SPAP = Systolic pulmonary artery pressure; EuroSCORE = European System for Cardiac Operative Risk Evaluation; CPB = Cardiopulmonary bypass

 No significant differences were seen between groups in any of the preoperative and intraoperative patient characteristics.

###  Primary Endpoint

 Hemodynamic parameters were kept stable (all achieved treatment target values) throughout the observation period. However, the mean arterial pressure at 5 minutes after heartbeat recovery (T_2_) and the end of CPB (T_3_) of group P-CPB were lower than those of group S-CPB (66.3 ± 4.8 mmHg and 70.7 ± 5.5 mmHg in group P-CPB versus 69.7 ± 5.7 mmHg and 76.3 ± 4.9 mmHg in group S-CPB, respectively) (*P* < 0.05). Cardiac index at the end of CPB (T_3_) and ScvO_2 _at 5 minutes after heartbeatrecovery (T_2_) of group P-CPB were also lower than those of group S-CPB (2.5 ± 0.5 L min^-1^ m^-2^ and 72.0 ± 13.5 % in group P-CPB versus 2.8 ± 0.5 L min^-1^ m^-2^ and 78.6 ± 8.6 % in group S-CPB, respectively) (*P* < 0.05) (Table 2).

**Table 2:** Hemodynamic data during CPB

**Parameter**	**Group S-CPB (** ***n*** **=27)**	**Group P-CPB (** ***n*** **=28)**	***P*** ** value**
MAP (mmHg)			
T_1_	70.4 ± 6.1	69.5 ± 6.0	0.582
T_2_	69.7 ± 5.7	66.3 ± 4.8	0.020
T_3_	76.3 ± 4.9	70.7 ± 5.5	0.000
CI (L min^-1^ m^-2^)			
T_1_	2.5 ± 0.5	2.5 ± 0.6	0.929
T_2_	2.6 ± 0.5	2.4 ± 0.5	0.348
T_3_	2.8 ± 0.5	2.5 ± 0.5	0.023
SVRI (d.s cm^-5^ m^-2^)			
T_1_	2022 ± 574	2048 ± 531	0.862
T_2_	1916 ± 504	1901 ± 459	0.909
T_3_	1884 ± 416	1868 ± 446	0.891
ScvO_2_ (%)			
T_1_	73.9 ± 8.9	70.1 ± 9.8	0.137
T_2_	78.6 ± 8.6	72.0 ± 13.5	0.038
T_3_	75.4 ± 6.4	71.6 ± 7.7	0.053
Lactate (mmol L^-1^)			
T_1_	1.9 ± 1.0	1.8 ± 1.2	0.661
T_2_	3.1 ± 1.1	3.1 ± 1.2	0.935
T_3_	4.0 ± 1.5	4.2 ± 1.6	0.692

Data are presented as mean ± SD. CPB = Cardiopulmonary bypass; MAP = Mean arterial pressure; CI = Cardiac index; SVRI = Systemic vascular resistance index; ScvO_2_ = Central venous oxygen saturation; T_1_ = Just before the start of CPB; T_2_ = 5 minutes after heart beat recovery; T_3_ = The end of CPB

 Need for inotropes and vasopressors was significantly different between groups. The proportion of patients using dobutamine and noradrenaline during CPB of group S-CPB were lower than those of group P-CPB (18.5 % and 14.8 % in group S-CPB versus 46.4 % and 39.3 % in group P-CPB, respectively) (*P* < 0.05) (Table 3).

**Table 3:** Need for inotropes and vasopressors during CPB

**Parameter**	**Group S-CPB (** ***n*** **=27)**	**Group P-CPB (** ***n*** **=28)**	***P*** ** value**
Dobutamine n (%)	5 (18.5)	13 (46.4)	0.027
Vasopressor n (%)			
Phenylephrine	26 (96.3)	26 (92.9)	1.000
Noradrenaline	4 (14.8)	11 (39.3)	0.042

CPB = Cardiopulmonary bypass.

###  Secondary Endpoints 

 Total complications were not different between groups S-CPB and P-CPB (66.7% versus 64.3%, *P* = 0.853).The rates of complications and mortality within 30 days after surgery of the two groups were not significantly different with *P* > 0.05 (Table 4). No significant differences were seen between groups.

**Table 4:** Morbidity and mortality within 30 days after surgery

**Parameter**	**Group S-CPB (** ***n*** **=27)**	**Group P-CPB (** ***n*** **=28)**	***P*** ** value**
Psychosis (n, %)	2 (7.4)	1 (3.6)	0.611
Myocardial ischemia (n, %)	1 (3.7)	2 (7.1)	1.000
Myocardial infarction (n, %)	0 (0.0)	0 (0.0)	NA
Pneumonia (n, %)	3 (11.1)	2 (7.1)	0.669
Renal injury (n, %)	0 (0.0)	2 (7.1)	0.491
Elevated transaminases (n, %)	13 (48.1)	13 (46.4)	0.898
Thrombocytopenia (n, %)	9 (33.3)	6 (21.4)	0.322
Death (n, %)	0 (0.0)	0 (0.0)	NA
Overall complication (n, %)	18 (66.7)	18 (64.3)	0.853

CPB = Cardiopulmonary bypass.

## Discussion

 This study highlights that the use of sevoflurane (instead of propofol) during CPB is effective in maintaining hemodynamic stability during CPB time (when compared with the control group) in patients undergoing cardiac surgery under CPB. Many factors influence hemodynamic instability during CPB in cardiac surgery patients. Of these, patient characteristics and surgery-related events such as surgery types and procedures, anesthesia protocols, CPB protocol, etc. were the most common causes. These characteristics of the two groups were similar, suggesting that the group using sevoflurane to replace propofol during CPB maintained stable hemodynamic parameters with less need for inotropes and vasopressors during CPB time than those in the control group were not caused by differences in patient characteristics and surgery-related events but instead that appears to be related to the choice of sevoflurane.^27,28^

 CPB is an essential element of cardiac surgical practice. The use of CPB technology allows cardiac surgical procedures to be performed smoothly in a motionless, bloodless surgical field.^24,29,30^ Despite significant advances in surgical techniques as well as anesthesia and CPB techniques, hemodynamic disorders during CPB are unavoidable.^3,24,31-34^ Hemodynamic disturbances during CPB are characterized by severe, vasopressor-resistant vasodilation due to activation of nitric oxide synthase, vascular smooth muscle ATP-sensitive potassium channels and relative deficiency of vasopressin.^24^ The choice of the appropriate anesthetic that has little effect on hemodynamics will contribute to hemodynamic stability during CPB, thereby reducing organ dysfunction caused by CPB and will contribute to reducing the rate of complications and mortality.^35^

 Animal studies have shown that sevoflurane has hemodynamic effects through effects on sympathetic nervous system activity, reducing myocardial contractility and systemic vascular resistance.^36,37^ However, sevoflurane has little or no effect on peripheral sympathetic nerve activity in young and healthy volunteers.^38^ Clinical outcomes in patients undergoing open heart surgery have also demonstrated the advantages of sevoflurane when compared with propofol in maintaining hemodynamic stability before and after CPB. But during CPB, the pharmacokinetics of anesthetics are altered due to hemodilution and altered metabolism leading to variable effect.^24,39^ Therefore, the effect of sevoflurane on the hemodynamic response during CPB may not be the same in the pre- and post-CPB periods. Currently, clinical data on the effect of sevoflurane on the hemodynamic response during CPB in patients undergoing open heart surgery, especially in patients undergoing valvular and congenital heart surgery, are lacking. And our research results have solved this problem. MAP at 5 minutes after heart beat recovery and the end of CPB as well as ScvO2 at 5 minutes after heart beat recovery and CI at the end of CPB of intervention group (group S-CPB) were higher than those of the control group (group P-CPB) and all achieved the treatment target values. Although the values of hemodynamic parameters during the observation period of both groups reached the treatment target values, these higher values ​​of the intervention group may be more conducive to improving tissue perfusion and oxygen supply for cardiac surgery patients.^40,41^ In addition, the proportion of patients using dobutamine and noradrenaline during CPB was also lower in group S-CPB. That means using sevoflurane instead of propofol for anesthesia during CPB time maintained stable hemodynamic parameters with less need for inotropes and vasopressors during CPB compared to the control group. The results of our study were in partial agreement with those reported by Essa et al as well as those of Nader et al both in patients undergoing CABG surgery under CPB.^15,16^ In the study by Essa et al the authors found that the use of sevoflurane during CPB resulted in a higher MAP with no difference in noradrenaline requirements during CPB when compared with propofol.^16^ Meanwhile, the study by Nader et al showed that the addition of sevoflurane to the cardioplegia solution during CPB resulted in reduced inflammatory response, improved cardiac function, and maintained stable hemodynamics.^15^ However, the results of our study did not agree with the report of Farwa et al^17^ also in patients undergoing CABG surgery. In the study by Farwa et al, it was shown that compared to propofol, anesthesia with sevoflurane during CPB resulted in higher MAP but also significantly higher noradrenaline requirements.^17^ We speculate that the reasons for the somewhat different findings in outcomes between our study and those of the above authors may be due to the difference in study subjects and drug administration for maintenance of anesthesia during CPB. Our study subjects included patients undergoing heart valve surgery and congenital heart surgery (atrial septal defect repair, ventricular septal defect repair) while the study subjects of the above authors were patients undergoing CABG surgery under CPB, so the effect of anesthetic drugs on hemodynamic response during CPB may not be the same. Regarding the drug administration during CPB, Essa et al as well as Farwa et al used 1 to 2% sevoflurane to maintain anesthesia in the intervention group and propofol at a rate of 75 to 125 μg kg^-1^ min^-1^ in the control group, while Nader et al added 2% sevoflurane to the cardioplegic solution in the intervention group and the control group used propofol alone at a rate of 100 to 150 μg kg^-1^ min^-1^.^15,16,17^ All of these drug administrations were different from those in our study. In our study, we used target-controlled infusion of propofol with effect site concentration (Ce) at 3 – 4 μg mL^-1^ to maintain anesthesia in the control group, while in the intervention group we maintained anesthesia by sevoflurane with a MAC of 1 ± 0.2. Furthermore, in both groups we used BIS to monitor the level of anesthesia, which could ensure adequate level of anesthesia for the patients as well as lead to less hemodynamic effects of anesthetics with different levels in both groups, and the results were somewhat different from the above studies.

 Blood lactate, a biomarker of circulatory failure, is often elevated during and after cardiac surgery and it is considered a predictor of major events related to morbidity and mortality.^3,42^ In this study, plasma lactate levels at the end of CPB in both groups increased compared to before CPB, but the difference between the two groups was not statistically significant. This shows that both groups had circulatory failure, but because the hemodynamics of both groups were maintained stable during CPB under the effect of inotropes and vasopressors, there was no significant difference between the two groups in terms of blood lactate concentration. Our results were similar to those of Essa et al^16^ as well as Farwa et al^17^ as mentioned above.

 In addition, the effectiveness of anesthetics is also shown in reducing complications and mortality after cardiac surgery under CPB. There are many factors that affect complications and mortality in patients undergoing cardiac surgery under CPB, such as individual characteristics (gender, weight), type and technique of surgery, duration of CPB, aortic cross-clamp time, etc. and blood lactate levels.^27,28^ In this study, the characteristics of the study patients, the surgical, anesthetic and CPB characteristics of the two groups were quite similar that helped to eliminate the confounding effects of these factors in the evaluation of secondary endpoints. In particular, the blood lactate concentrations of the two groups were not different. That explained why complications and mortality rates within 30 days after surgery of the sevoflurane group were not significantly different from those of the control group. The results of our study were similar to those of some other authors in patients undergoing CABG and heart valve replacement surgery.^7,10^ However, meta-analyses in large numbers of patients undergoing cardiac surgery under CPB suggested that sevoflurane was associated with reduced complications and mortality in patients undergoing cardiac surgery under CPB.^43^ Therefore, longer studies with larger sample sizes are needed to resolve this issue.

 There are also several limitations of the present study. First, a double-blind study was not conducted because anesthesiologists could not be blind to the anesthetic technique used in each group; only the part related to statistical analysis was blinded. Second, this study is not representative of all subjects undergoing cardiac surgery under CPB because it included only patients undergoing heart valve surgery and congenital heart surgery (atrial septal defect repair, ventricular septal defect repair). Third, we only calculated the study sample size based on the CI at the end of CPB of a previous pilot study and not on additional complications and mortality. And that may be the reasons why we could not demonstrate a significant difference in complications and mortality within 30 days after surgery even though group S-CPB was hemodynamically stable with less need for inotropes and vasopressors during CPB than group P-CPB. Therefore, a study with larger sample sizes based on hemodynamic parameters during CPB as well as complications and mortality within 30 days after surgery are needed to clarify these issues.

## Conclusion

 In conclusion, in patients undergoing cardiac surgery under CPB, the use of sevoflurane in place of propofol for anesthesia during CPB results in hemodynamic stability with less need for inotropes and vasopressors during CPB but morbidity and mortality within 30 days after surgery were not significantly different when compared with the control group.

## Competing Interests

 All authors have no conflict of interest to declare.

## Ethical Approval

 The ethics committee of 108 Military Central Hospital approved this study design (ref. 275/QD-V108) and written informed patient consent was obtained. The study was conducted according to the Declaration of Helsinki.
